# Mycophenolic acid area under the curve recovery time following rifampicin withdrawal

**DOI:** 10.4103/0971-4065.62091

**Published:** 2010

**Authors:** V. M. Annapandian, D. H. Fleming, B. S. Mathew, G. T. John

**Affiliations:** Department of Nephrology, Christian Medical College, Vellore, India; 1 Department of Clinical Pharmacology, Christian Medical College, Vellore, India

**Keywords:** Mycophenolate, rifampicin, interaction, renal transplantation

## Abstract

Renal transplant patients prescribed mycophenolate mofetil (MMF) may require treatment for tuberculosis with a regimen including the tuberculocidal drug rifampicin. MMF is an ester prodrug which is rapidly hydrolysed to the active compound, mycophenolic acid (MPA). Therapeutic drug monitoring of mycophenolate involves the measurement of MPA area under the curve (MPA-AUC_0-12_). Rifampicin is known to increase the metabolism and decrease enterohepatic recirculation of mycophenolic acid, (MPA). When MPA is monitored after the discontinuation of rifampicin, an important factor is the time required for the MPA area under the curve to return to the pre-rifampicin value. At present this is not known. This report describes one such renal allograft patient, on long term MMF and prescribed rifampicin by a local physician. As expected there was a clinically significant decrease in MPA-AUC_0-12_ Three weeks after rifampicin was discontinued the MPA-AUC_0-12_ was still only 65% of the pre-rifampicin value and only 55% of the steady state MPA-AUC_0-12_ measured six months later.

## Introduction

Mycophenolate, an immunosuppressant widely prescribed in renal transplantation,[1] is available as mycophenolate mofetil (MMF). Given orally, it is rapidly absorbed and converted to the active moiety, mycophenolic acid (MPA). MPA is metabolized in the intestine, liver and kidney by enzymes of the uridine glucuronosyl transferase system[2] to its main metabolites MPA 7-O-glucuronide (MPAG) and MPA acyl-glucuronide (AcMPAG). MPA shows enterohepatic re-circulation with a secondary concentration peak commonly occurring between 6 and 12 hours post-dose. Individualization of drug dosage is based on the maintenance of the MPA area under the curve (MPA-AUC_0-12_) within 30-60 mg.h/L.[3] Therapeutic drug monitoring (TDM) of mycophenolate is still controversial but is advocated owing to the large inter-individual variability in pharmacokinetic profiles[4] and intra-individual variability between early and stable transplant periods.[5,6] In the Clinical Pharmacology unit of this hospital, monitoring of total MPA involves the calculation of MPA-AUC_0-6 hrs_ from 10 plasma specimens collected over six hours and corrected mathematically to give a corrected dose-interval MPA-AUC (AUC_0-12corr_)[7] This AUC has an intra class correlation (ICC) ≥0.97 with the total measured 12-hour AUC.[7]

Rifampicin is a commonly used tuberculocidal drug known to increase metabolism and decrease enterohepatic re-circulation of MPA.[8] However, the time required for the MPA-AUC_0-12_ to return to the pre-rifampicin value in renal allograft patients is, to our knowledge, unknown. This case report describes a clinically significant decrease in MPA-AUC when rifampicin was administered for two weeks to a renal allograft recipient on long term MMF therapy, and the evidence of a continued induction effect 3 weeks after the withdrawal of rifampicin.

## Case Report

A 57-year-old male renal allograft recipient in 2003 was prescribed prednisolone, MMF (1,500 mg daily) and losartan, therapeutic drug monitoring of MPA was performed twice in 2007, on both occasions, the patient was in steady state with respect to MPA pharmacokinetics and the biochemistry was normal. The MPA AUC_0-12corr_ was 42.9 mg.h/L in April and 54.3 mg.h/L in October 2007.

The patient was from a neighbouring country and returned home. Whilst there, in early December 2007, he was diagnosed with pulmonary tuberculosis and started on anti-tubercular therapy which consisted of rifampicin, isoniazid, ethambutol, pyrazinamide and pyridoxine. Two weeks later, the patient presented to our transplant unit with mild elevation of creatinine compared to the previous visit (2.0 mg% versus 1.7 mg%). According to the protocol for the management of tuberculosis in transplant patients in our transplant unit rifampicin was discontinued and ofloxacin was started. Mycophenolate was continued at the same dose. One week after rifampicin was discontinued MPA was monitored and the MPA-AUC_0-12corr_ was 13.9 mg.h/L. This is a 74.4% reduction in MPA-AUC_0-12corr_ compared to the previous measurement (13.9 vs 54.3 mg.h/L in October).

Three weeks after discontinuation of rifampicin the MPA-AUC_0-12corr_ was again measured and had increased to 35.3 mg.h/L. This is a 2.5 fold increase in the MPA-AUC_0-12corr_ (13.9 to 35.3 mg.h/L) from two weeks earlier however, it is only 65% of the MPA-AUC_0-12corr_ prior to rifampicin. The serum creatinine had improved to 1.7 mg%. The patient was unable to stay longer and returned home.

**Figure 1:**
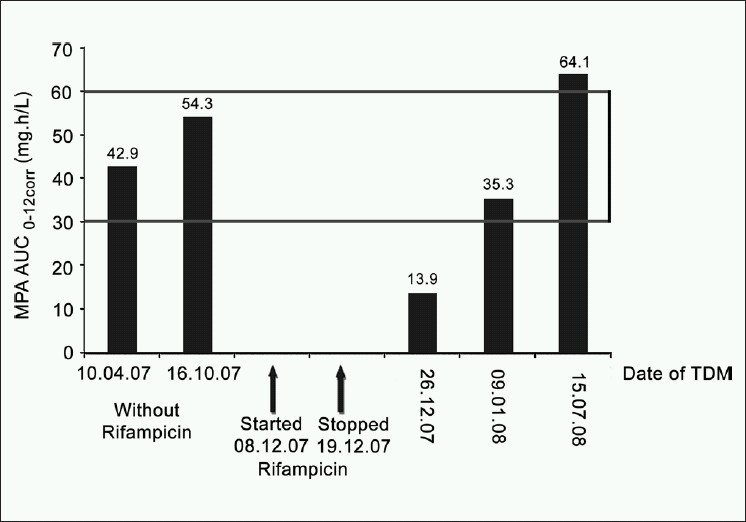
Influence of rifampicin on mycophenolic acid area under the curve over the time

In July 2008 when the patient re-visited the transplant unit, MPA-AUC_0-12corr_ was again measured and 64.1 mg.h/L. Serum albumin was within the acceptable range (3.4 to 4.7 gm/dl) throughout the period of MPA monitoring. Figure 1 shows the time course of rifampicin treatment and TDM results.

## Discussion

Rifampicin was co-administered with MPA for a relatively short time in this patient and MPA-AUC_0-12corr_ measured one week after discontinuing the rifampicin showed a continued clinically significant effect of rifampicin on the pharmacokinetics of MPA. Kuypers et al.[8] first reported this marked reduction in MPA exposure with rifampicin in a patient on long term rifampicin therapy and prescribed MMF. Naesens *et* al.[9] also found a significant reduction in total MPA-AUC_0-12_ after only eight days of rifampicin co-administration. Naesens and co-workers[9] investigating rifampicin as a probe drug on MPA pharmacokinetics, reported changes in MPA kinetics due to induction of the glucuronidation enzyme activity and decreased enterohepatic recirculation. They reported that their patients showed no signs of toxicity despite the increased rate of Ac-MPAG formation and no apparent alteration in its clearance. In a similar way the patient reported here did not show any signs of toxicity. However, in both cases this could be due to the short period over which rifampicin was administered.

Kuypers et al.[8] reported that in their patient, who had received a combined heart-double-lung allograft, the induction effect of rifampicin ceased approximately two weeks after discontinuation of the drug. In our patient three weeks after the discontinuation of rifampicin, there was a 2.5-fold improvement in MPA-AUC_0-12corr_ compared to one week after rifampicin was stopped however, the MPA AUC was still only 65% of that prior to the introduction of rifampicin. As this patient was from another country and needed to return home a further MPA-AUC could not be performed until after 6 months. The value of the MPA-AUC_0-12corr_ in July 2008 confirms that the AUC continued to rise, as the MPA-AUC three weeks following rifampicin withdrawal was significantly lower than the final steady state MPA-AUC values. A dosage change based on MPA- AUC measurement at this time would be incorrect.

## Conclusion

This case confirms that a relatively short exposure to rifampicin significantly reduces the MPA-AUC and that it may require more than three weeks for pre-rifampicin concentrations to be restored. Care should be taken by physicians following rifampicin withdrawal, even after short exposure, to allow adequate time prior to making dosage adjustments based on MPA-AUC measurements.
